# Call for Consensus in the Evaluation of Circulating Matrix Metalloproteinases in Chagas Disease

**DOI:** 10.4269/ajtmh.21-0860

**Published:** 2022-07-05

**Authors:** Norma L. Bautista-López, Richard Schulz

**Affiliations:** ^1^ Macdonald Campus, Institute of Parasitology, McGill University, Montreal, Quebec, Canada;; ^2^ Departments of Pediatrics and Pharmacology, Cardiovascular Research Centre, Mazankowski Alberta Heart Institute, University of Alberta, Edmonton, Alberta, Canada

## Abstract

Infection with the *Trypanosoma cruzi* parasite is endemic in parts of America. Approximately 30% of people infected develop Chagas cardiomyopathy, the most common cause of heart failure in these regions. No suitable biomarker that reflects the evolution of the disease has been widely accepted as of yet. There is substantial evidence, however, of a strong inflammatory reaction following infection with *T. cruzi* that could activate matrix metalloproteinases (MMPs). Emerging research suggests the involvement of MMPs in Chagas cardiomyopathy and there is a growing interest in measuring the blood levels of MMPs as diagnostic and/or prognostic indicators of heart damage in Chagas patients. This perspective discusses the lack of consensus on the best method for MMP evaluation. Some studies are based on MMP concentrations and activities in serum whereas others use plasma. We believe that these different methods of evaluation have led to incongruent and poorly comparable data on the blood levels of MMPs in Chagas patients. A standard for the preparation of blood samples needs to be adopted for the study of MMPs as markers of Chagas cardiomyopathy to ensure better comparability of research results.

## CHAGAS DISEASE

This is a debilitating parasitic disease that is prevalent in parts of America. It was estimated that 6.2 million people had Chagas disease in 2017, mostly in Mexico, Central, and South America,^1^ resulting in an estimated 7,900 deaths.^2^ It is characterized by an acute phase, which occurs after infection with *Trypanosoma cruzi*, followed by a chronic phase. The chronic phase begins with an asymptomatic period, after which 20–30% of infected people develop cardiomyopathy over the course of 10–30 years.^3^^,^^4^

The pathophysiological mechanisms leading to Chagas cardiomyopathy are under study by several research groups. *Trypanosoma cruzi* is often found in the striated muscle fibers of the heart during the initial stages of the disease.^5^ There is substantial evidence of a strong inflammatory reaction during *T. cruzi* infection that is triggered by persistent parasitemia.^6^ The parasitic load in the chronic stage is low, however, fibrous tissue and fat replace damaged cardiac muscle tissue and inflammatory foci form, composed primarily of T cells and macrophages, with a few eosinophils, plasma cells, and mast cells.^5^ Inflammation is an important trigger to induce the production and/or activity of matrix metalloproteinases (MMPs); in addition the MMPs are inherent mediators of inflammation.^7^

The antiparasitic drugs currently available for Chagas disease treatment are benznidazole and nifurtimox. These drugs are highly recommended for use in the acute phase, but their use in chronic stages remains controversial due to significant toxicity profiles and their unproven role in preventing cardiomyopathy.^8^^,^^9^ New pharmaceutical approaches must be investigated to reduce heart damage.

## MMPs AND THEIR MEASUREMENT

In humans, MMPs comprise 23 structurally related enzymes that are part of a large family of enzymes called proteinases, which cleave substrate proteins both inside and outside of cells.^10^ The MMP domain structure consists of signal peptide, amino terminal propeptide, catalytic domain with Zn^2+^ binding site, and carboxy terminal domains. Matrix metalloproteinase activity is regulated by endogenous tissue inhibitors of metalloproteinases (TIMPs). Several pathological conditions can be caused by the disruption of the balance between MMPs and TIMPs.^11^ Matrix metalloproteinase activity, especially that for MMP-2 and MMP-9, can be estimated with gelatin zymography, a widely used technique,^12^^,^^13^ which separates and identifies MMP-2 and MMP-9 isoforms by their molecular weights. Matrix metalloproteinase concentrations have also been measured using ELISA. In this immunoassay, the MMP binds to a capture-specific antibody and is then detected with a biotinylated detection antibody. Matrix metalloproteinase concentrations have also been measured with multiplex analysis, a derivative of ELISA in which magnetic beads are used to bind the capture antibody. This assay can be used to measure multiple MMPs simultaneously. Unfortunately, there is no gold standard to measure MMP levels or activity. However, to understand the role of MMPs in physiological or pathological processes, reliable methods to assess MMPs are mandatory.^14^

## MMPs AS MODULATORS OF INFLAMMATION

Inappropriate activation of MMPs results in cardiac remodeling and enlargement, which can lead to heart failure.^15^ As well, MMP-2 exhibits several unique intracellular actions, including proteolysis of specific sarcomeric proteins in heart muscle cells that diminishes the contractile efficiency of heart muscle.^16^ Matrix metalloproteinase-2 was activated in the myocardium of isolated rat hearts exposed to proinflammatory cytokines, causing contractile dysfunction which was abrogated by MMP inhibitors.^17^ In vitro studies of human monocytes showed that interleukin-1 beta or tumor necrosis factor-alpha increased MMP-9 production and activity.^18^ In preclinical models of myocardial infarction, inhibition of MMPs after infarction led to the preservation of left ventricular structure and diastolic function.^19^ In mice infected with *T. cruzi*, an MMP inhibitor significantly decreased heart inflammation in the acute phase.^20^

Accumulating evidence suggests the involvement of MMPs in Chagas cardiomyopathy and there is a growing interest in measuring the blood levels of MMPs as diagnostic and/or prognostic indicators of heart damage in Chagas patients.^21^^–^^26^ There is a lack of consensus, however, on the best method for evaluating MMPs. For example, MMP concentrations and activities differ between serum^22^^–^^25^ and plasma.^21^^,^^26^

## SAMPLE COLLECTION AND PREPARATION

Serum is the part of the blood that remains liquid after coagulation and clotting factors, erythrocytes, leukocytes, and platelets have been removed. To collect serum, blood samples are allowed to coagulate, a process in which the blood-borne cells and platelets aggregate by clot formation. Most serum collection tubes contain clot accelerators such as soluble silica particles, kaolin granulates, or silica gel; therefore, serum lacks fibrinogen and all clotting factors.

To obtain plasma from whole blood, the use of anticoagulants is necessary to prevent clotting and to separate plasma from all types of blood cells and platelets. During its preparation, in contrast to serum, the cells remain suspended and fibrinogen and clotting factors are still present. The most common anticoagulants are ethylenediaminetetraacetic acid and sodium citrate, which bind calcium and thereby inhibit specific pathways of the coagulation cascade. In 1998, Jung and collaborators reported differences between MMP concentrations in plasma and serum.^27^ It was only later, however, that the effect of preparing serum or plasma on MMP activity profiles was evaluated by zymography. Makowski et al.^28^ performed gelatin zymography analysis of sera and plasma samples prepared from the blood of five healthy participants. They did not find that the concentration of 72 kDa MMP-2 differed significantly between serum and plasma. However, MMP-9 was significantly increased in serum versus anticoagulated plasma.^28^ They suggested that the release of MMPs can be minimized by collecting plasma from blood using citrate as an anticoagulant and by processing the samples in a timely fashion.^28^ Mannello et al.^29^ did not observe any difference in 72 kDa MMP-2 proteolytic activity in serum versus plasma when they performed gelatin zymography on samples from 20 healthy participants. However, they reported that MMP-9 isoforms were two to 10 times higher in serum versus plasma.^29^ Jung et al. used immunoassays and observed that serum MMP-1, -8, and -9, and TIMP-1 concentrations were higher compared with plasma samples.^30^^–^^32^

When whole blood clots in the sample tube, MMPs are released due to the degranulation of leukocytes and platelets.^33^ Fernandez-Patron et al.^34^ suggested that elevated MMP-9 levels come from leukocytes and platelets during coagulation and fibrinolysis. Elevated MMP-9 concentrations in serum, therefore, primarily reflect the release of proteases by leukocytes and platelets during the clotting process in the blood collection tube.^35^ Moreover, platelets not only express MMP-1, MMP-2, and MMP-9, but these MMPs are translocated to the membrane surface and released upon activation.^34^^,^^36^^–^^38^ Mannello et al.^39^ proposed that silicate favors a stable gelatinase conformation in association with water and that this increases enzyme activity without loss of its autoinhibitory prodomain. The concentration of MMP-9 in serum collected in tubes with clot activator were reported to be three times higher than in serum samples without it.^40^ Studies have shown that MMP-9 levels can be 4- to 10-fold higher in serum than in plasma.^32^^,^^42^ In samples obtained during colorectal cancer screening, MMP-9 levels were reported to be more than 5-fold times higher in serum than in citrate plasma.^41^

**Figure 1.**
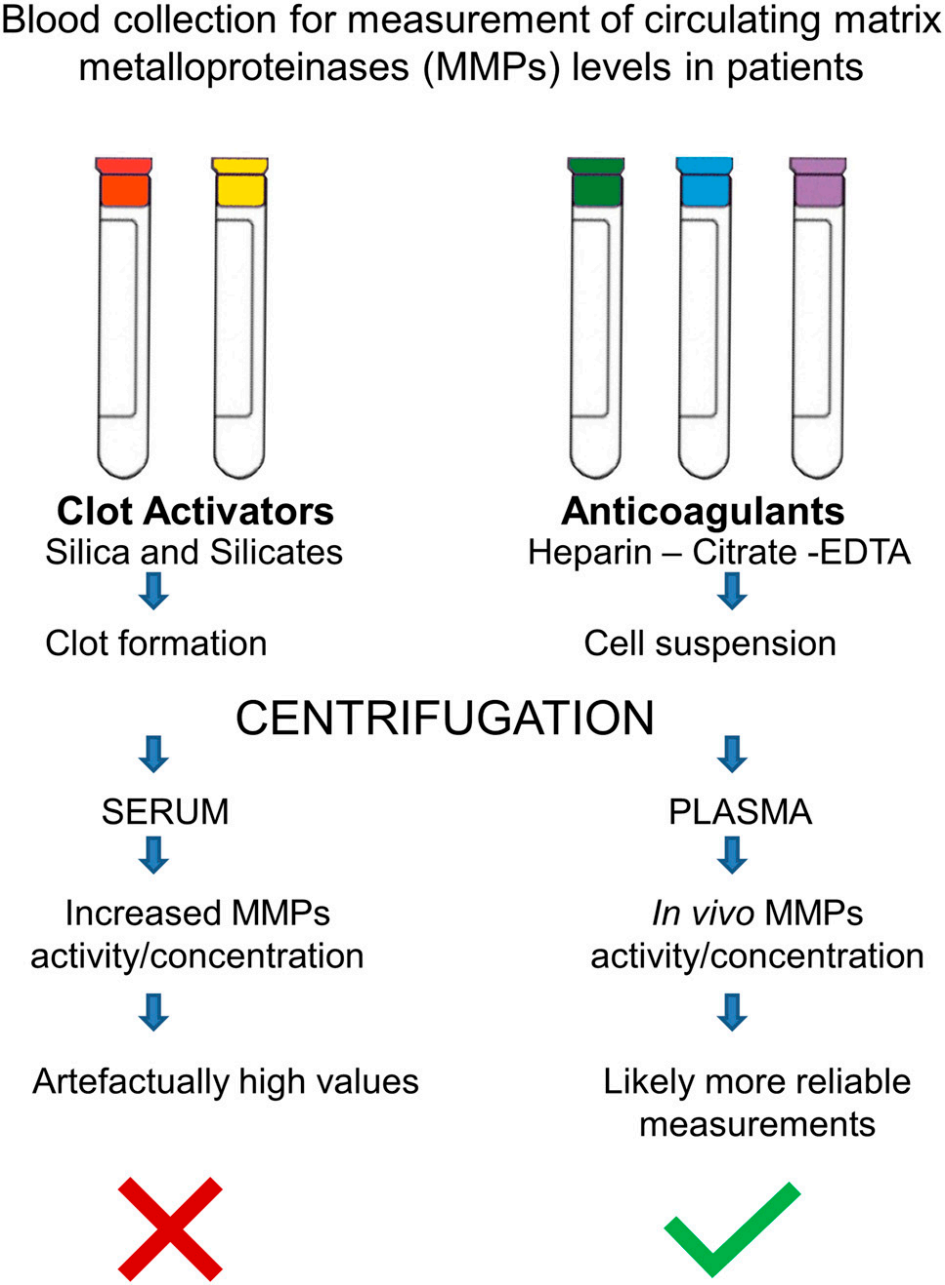
Blood collection for measurements of circulating matrix metalloproteinase (MMP) levels in patients. When clot activators are used for isolating serum, MMPs are released and activated due to degranulation of leukocytes and platelets. As a result, artefactually increased concentration and activity of MMPs are measured. Instead, when plasma samples are obtained by the use of anticoagulants, the MMPs activity/concentrations better reflect in vivo circulating levels, and should be more comparable between studies. This figure appears in color at www.ajtmh.org.

Given the comparisons in different conditions, among others not mentioned here, we suggest that it is necessary to standardize blood collection practices for reproducible and comparable determination of MMP levels in clinical and preclinical studies. We suggest that it is of considerable importance, for instance, to avoid erroneously high measurements of MMP levels by using plasma and not serum^27^^–^^42^ (Figure 1).

## MMPs IN CHAGAS DISEASE

Matrix metalloproteinases levels in circulating blood were studied as a possible tool to prognosticate heart damage in Chagas disease patients. Some groups analyzed MMP levels in serum^22^^–^^25^ and others in plasma,^21^^,^^26^ with conflicting results. We^21^ suggested that plasma MMP-2 and -9 be measured as biomarkers of the early progression of Chagas cardiomyopathy. This cross-sectional study involved 188 Colombian participants classified for heart failure severity according to the New York Heart Association guidelines and showed a significant increase of circulating MMP-2 and MMP-9 activities in plasma from individuals who were seropositive for *T. cruzi*, as compared with seronegative controls. This study also revealed higher plasma activities of MMP-9 in patients with electrocardiographic abnormalities and in those with dilated Chagas cardiomyopathy. Conversely, Fares et al.^22^ analyzed serum MMP-2 and -9 activities in a study of 27 participants classified for heart failure according to the New York Heart Association and found that only MMP-9 levels increased proportionally to the degree of Chagas disease while there was no observable change in MMP-2 activities. Okamoto et al.^23^ used multiplex analysis of serum samples (participants classified by the New York Heart Association) to show that the MMP-2/MMP-9 ratio increased with the severity of cardiac disease, where patients with severe cardiac Chagas disease presented with increased levels of MMP‐9 compared with MMP‐2. Clark et al.^24^ studied a cohort of participants classified by the American College of Cardiology/American Heart Association guidelines. They observed different patterns of response in MMP-2 levels in serum samples as compared with the two aforementioned studies.^22^^,^^23^ They performed a cross-sectional multiplex analysis of 64 individuals and observed that levels of MMP-2 in serum increased with the severity of Chagasic cardiomyopathy. Sherbuk et al.^25^ used multiplex assays of sera to study 409 participants classified by the New York Heart Association guidelines and found MMP-2 concentration to be predictive of mortality. More recently, Medeiros et al.^26^ used multiplex immunoassays of MMP-2 and MMP-9 in plasma samples from 28 Chagas patients classified by the Latin American guidelines for the diagnosis and treatment of Chagas heart disease and the New York Heart Association guidelines. Even from this small cohort, they were able to identify MMP-2 as a biomarker for fibrosis replacement in early remodeling and MMP-9 as a marker of late fibrosis and severe cardiac remodeling.

## PROPOSAL FOR CONSENSUS

We believe that the results of some studies of blood levels of MMPs in Chagas patients differ because of the use of either plasma or serum, and the methods and materials used. The literature suggests that the use of plasma gives more reliable results than the use of serum.^21^^,^^26^ Regarding methodology, there is no gold standard to measure MMP levels or activity. Gelatin zymography, a widely used technique, can be used to detect and distinguish between MMP-2 and MMP-9 and estimate their activities.^12^^–^^14^ Although zymography is more laborious than ELISA, the latter cannot be used to distinguish between latent and active MMPs.^12^^,^^14^ One advantage of ELISA is the possibility to measure MMPs and other proteins simultaneously if the assays are performed on a multiplex reader, but multiplex ELISA kits are very expensive. We suggest that gelatin zymography could be used to measure circulating MMP activities in plasma from Chagas patients and the results compared with those from the use of immunoassays, since this comparison should be valuable for diagnosis and prognosis.

We, therefore, suggest that plasma measurements of MMPs be adopted as the standard in the study of MMPs as markers of Chagas cardiomyopathy, to ensure the comparability of results. Given the unjustifiably limited funding for research of neglected tropical diseases such as Chagas disease, researchers should give priority to prepare samples and perform tests that are comparable to other research groups. A consensus on the most efficacious kind of blood sampling and MMP measurements should result in data that are more scientifically rigorous and informative.
